# Legacies of Hate: The Psychological Legacy of the Ku Klux Klan

**DOI:** 10.1177/01461672241292524

**Published:** 2024-12-16

**Authors:** Maximilian A. Primbs, Margaux N. A. Wienk, Rob W. Holland, Jimmy Calanchini, Gijsbert Bijlstra

**Affiliations:** 1 Radboud University, Nijmegen, The Netherlands; 2 Columbia University, New York, NY, USA; 3 University of California, Riverside, USA

**Keywords:** Ku Klux Klan, implicit bias, Bias of Crowds

## Abstract

The second coming of the Ku Klux Klan popularized the Klan and its ideas in the early 1920s, terrorizing Black American, their allies, and others deemed un-American. This article investigates the extent to which the cultural legacy of racial hatred of the Klan has persisted over the years. We use data from large online databases, multiverse analyses, and spatial models to evaluate whether regions with more historical Klan activity show higher levels of modern-day racial bias, and more modern-day White Supremacist activity. We find that regions with more Ku Klux Klan activity in the 1920s show higher levels of modern White Supremacist activity but, unexpectedly, lower levels of modern implicit and explicit racial bias. We discuss the implications of these findings for models linking historical events with present-day attitudes and behavior, and for situational models of bias more broadly.

Founded in 1865, the Ku Klux Klan might be the oldest still active terror movement in the United States (Bordewich, 2023). With White hoods, burning crosses, and midnight rallies, the Klan engaged in intimidation, bombings, and lynchings, often targeted Black Americans, and their allies. At its peak, the Klan featured several million members, de-facto ruled entire counties, and was supported by large parts of the population (Southern Poverty Law Center, 2011). Today, the Klan itself has faded mostly into obscurity with 3,000 to 8,000 members remaining nationwide (Anti-Defamation League, 2016). Yet, the hateful ideas it spread may still live on today. In this article, we investigate whether the cultural legacy of the Klan perpetuated itself within geographical regions and still shapes people’s minds and behavior today.

We ground our research in situational models of (implicit) bias (Calanchini et al., 2022; Payne et al., 2017; Payne & Hannay, 2021). These models argue that implicit racial bias is a product of, and endures in, the environments we live in—the physical environment we can see when walking down the street, but also the social and cultural environment, the norms, laws, and cultural habits guiding our interactions with the world. From this perspective, implicit racial bias is thus present not only within people but also within environments or situations (Payne et al., 2017; Payne & Hannay, 2021). Many features of environments are also inherently more long-lived and stable than individuals—be it Confederate monuments perpetuating White Supremacy for hundreds of years or the residuals of long-gone segregation policies still shaping neighborhoods today. Proponents of situational models thus argue that implicit racial bias reflects systemic racism (Payne & Hannay, 2021).

If implicit racial bias reflects systemic racism, then the legacy of historical inequality should be detectable in implicit bias scores of present-day populations (Payne et al., 2019). Accordingly, recent studies on the impact of historical inequalities on implicit bias have shown that various historical events or inequalities and present-day reminders of those inequalities are associated with increased levels of modern implicit bias (Payne et al., 2019; Vuletich et al., 2024; Vuletich & Payne, 2019). For example, Payne and colleagues (2019) investigated the relationship between the proportion of slaves in a region in 1860 and modern implicit bias and found that places with higher proportions of slaves in 1860 have greater pro-White implicit bias among White residents today. Similarly, Vuletich and colleagues (2024) showed that regions with a higher proportion of Black people during the Great Migration, during which Black Americans from the South migrated to Northern states, show greater pro-White implicit bias among current White residents. Together, these studies demonstrate that the legacy of historically significant events can still be detected today—in the form of geographically aggregated implicit bias.

Not only do aggregated implicit bias scores correlate with historical events, but they also correlate with regional behaviors and other outcomes. For example, Hehman and colleagues (2018) showed that higher levels of implicit bias in an area predict higher levels of disproportionate use of lethal force against Black people, and Stelter and colleagues (2022) found that higher levels of implicit bias in a region are associated with increased disproportionate stopping of Black drivers (see also Ekstrom et al., 2022; Payne & Rucker, 2022). Importantly, the police officers who disproportionately kill Black people or stop Black drivers are not necessarily the same people who completed the implicit bias tests. However, test takers and police officers share the same environment, which suggests that the implicit biases perpetuated in an environment are related to the behavior of people in that environment.

In this article, we test situational models of implicit bias in yet a different domain. That is, we argue that the Ku Klux Klan and the terror and hatred it spread left a lasting cultural and historical legacy in the United States, such that Klan activity in a given geographic region continues to shape biases and behavior today. We hereby expand situational models of bias by arguing that implicit bias is the means through which hate and hateful behavior persists within regions throughout the centuries. We test our claims by combining a historical database on the second coming of the Ku Klux Klan^1^ (1915–1940; Kneebone, 2015; Kneebone & Torres, 2023) with a large-scale database on implicit bias (Project Implicit; Xu et al., 2022) and a database on present-day White Supremacist activity (Anti-Defamation League, 2024), thus assessing historical inequalities, attitudes, and behavior in a single study.

In line with situational models of implicit bias and past research on the impact of historical inequalities on implicit bias, we predict that more county-level Klan activity in the past is associated with higher modern county-level implicit bias (Payne et al., 2017, 2019; Payne & Hannay, 2021; Vuletich et al., 2024). Concretely, we expect that regions with Klan klaverns (i.e., local chapters) in the past will have higher levels of modern implicit bias compared with regions with no klaverns (H1), and that regions with more historical klaverns will have higher levels of present-day implicit bias (H2). Moreover, given the links between aggregate-level implicit bias and behavior (Hehman et al., 2018; Stelter et al., 2022), we expect that regions with higher levels of modern implicit bias will have higher levels of modern White Supremacist activity (H3). Finally, we argue that the Klan’s legacy of hate also shapes behavior to this day. Hence, we expect that regions that had Klan klaverns in the past (H4), or regions with a higher number of klaverns (H5), will have a higher number of White Supremacist events today compared with regions with no or fewer klaverns.

To foreshadow, we do not find evidence for our pre-registered hypotheses that historical Ku Klux Klan presence is linked to higher levels of implicit bias. Instead, across many analyses and datasets, we find that historical Ku Klux Klan presence is robustly linked to lower levels of implicit racial bias, which forces us to think about bias and systemic inequalities in a completely novel manner.

## Method

### Transparency and Openness

The present study makes all processed data and analysis code freely available on the Open Science Framework (https://osf.io/h5sx6/?view_only=3f8cc0ff314f4410979f91e1b6f4be67). The unprocessed data and study materials are freely available on Project Implicit (https://osf.io/y9hiq/) and interested readers can view the implemented tests on https://implicit.harvard.edu/implicit/. The study, including hypotheses and analysis code, was pre-registered (https://osf.io/g3xru?view_only=cbf4ea078ad24bf09d0d8af89e1f3980 and https://osf.io/u3mbx/?view_only=8816ae9739134460ac071da5a5610615). We clearly report in the “Results” section which analyses are pre-registered and which analyses are not pre-registered. We report all data exclusions, manipulations, and all measures in the sample. Our study relies on existing data, hence the sample size was determined by the available data.

### Datasets and Measures

Our study made use of the 2004 to 2022 subset of the evaluative race implicit association test (IAT; Greenwald et al., 1998) data available on Project Implicit (Xu et al., 2022). The Project Implicit data are described in detail in the works of Charlesworth and Banaji (2019, 2021, 2022). We pre-registered the same inclusion criteria as in Primbs, Holland, and colleagues (2026) and for theoretical precision restricted our analyses to completed sessions of White participants currently based in the United States. Moreover, we removed all participants who reported more than one gender identity or who responded faster than 300 ms on 10% or more of the IAT (Greenwald et al., 2003). We added a new exclusion criterion by removing all participants who did not provide information on the county they lived in. These exclusion criteria were pre-registered (https://osf.io/g3xru?view_only=cbf4ea078ad24bf09d0d8af89e1f3980). Finally, for the purpose of facilitating geospatial analyses, we limit our analyses to the contiguous United States. This exclusion criterion was not pre-registered. The final sample size is 2,698,505 participants, 1,894,860 of whom are based in counties with a historical Klan klavern and 803,645 based in counties without a historical Klan klavern.

#### Implicit Bias

Implicit bias was measured with the evaluative race IAT (Greenwald et al., 1998). The race IAT is a reaction time measure in which participants categorize faces as African American (here referred to as Black) and European Americans (here referred to as White) and words as positive or negative (for a detailed description, see Primbs, Holland, et al., 2026). We assess bias in the IAT using the IAT D score (Greenwald et al., 2003). Higher IAT D scores are typically interpreted as higher levels of pro-White/anti-Black implicit bias. Although the reliability and validity of IAT have been heavily criticized in the context of individual differences (see Payne et al., 2017), regional aggregation addresses many of these criticisms (Hehman et al., 2019).

#### Ku Klux Klan

We obtained information about the presence and number of Ku Klux Klan klaverns from the “Mapping the Second Ku Klux Klan, 1919-1940” project (Kneebone, 2015; Kneebone & Torres, 2023). The dataset contains information about the rise and fall of the second Ku Klux Klan and is mostly based on contemporary newspaper articles. Readers can find more information about the project at https://labs.library.vcu.edu/klan/learn and in the accompanying paper (Kneebone, 2015). For the present project, we extracted the presence and number of Klan klaverns within a city from the dataset and subsequently matched each city with the county in which it is located. In some U.S. states, a city can be located in multiple counties. If a city was located in multiple counties, we asked R to assign the city to any one of these counties. This was to prevent us from making arbitrary judgments about what county a city predominantly belongs to.

#### White Supremacist Activity

We obtained information about White Supremacist activity from the Anti-Defamation League (2024) H.E.A.T. Map (Hate, Extremism, Antisemitism, Terrorism), which contains information about White Supremacist events and White Supremacist propaganda (here summarized as White Supremacist activity) in the United States. We used the 2017 to 2023 dataset downloaded in January 2024. This dataset contains data from 14.01.2017 to 31.07.2023 with a total of 25,613 incidents on a city level, 21,273 of which we could match to a county. If a city is located in multiple counties, we asked R to assign the city to any one of these counties. The database was created by the Center for Extremism of the Anti-Defamation League and is compiled based on newspaper articles, government reports, investigations, and other sources (see https://www.adl.org/resources/tools-to-track-hate/heat-map).

#### Control Variables

We included the population density and the proportion of Black people living within a county as control variables. Both measures reflect 5-year estimates from 2018 to 2022 from the American Community Survey (United States Census Bureau, 2024). We selected these control variables because we believe that both confound our effects of interest. The choice of control variables was pre-registered, and we report additional exploratory analyses with other plausible covariates in the results section.

## Results

### Confirmatory Analyses

**Figure 1.**
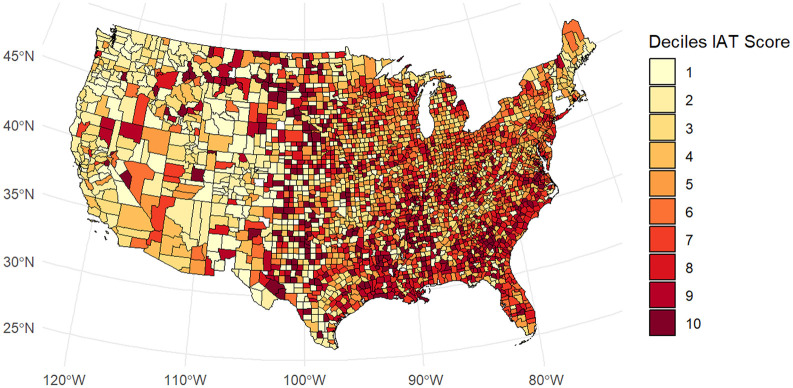
Implicit Bias Scores by County. Higher scores reflect stronger pro-White/anti-Black implicit bias.

**Figure 2.**
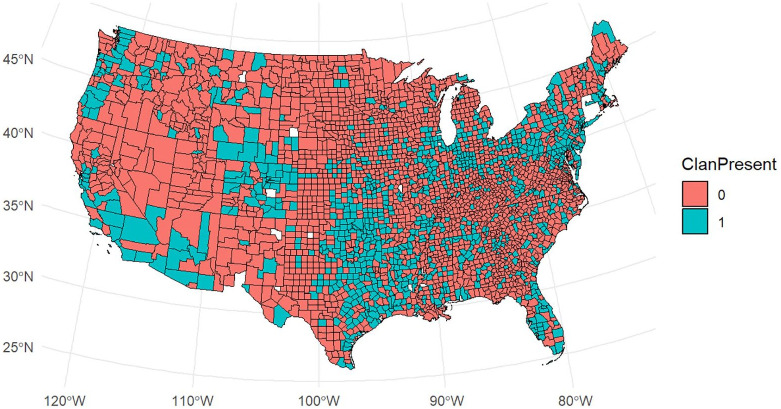
Klan Klaverns by County. “0” means no klavern and “1” means a klavern.

**Figure 3.**
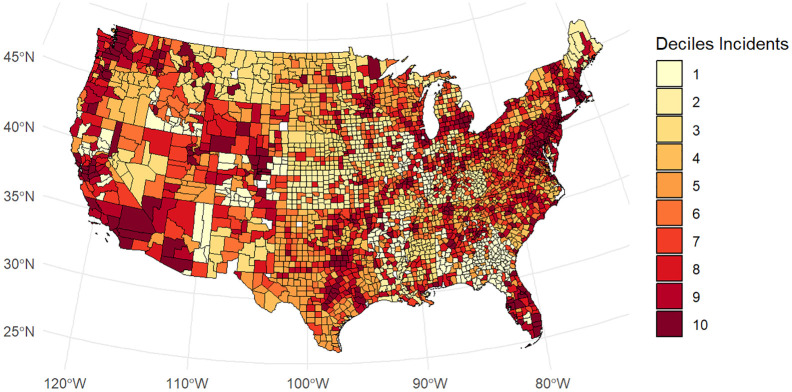
White Supremacist Activity by County. Higher values reflect more activity.

We pre-registered a set of confirmatory analyses for H1 to H5. For H1 and H2, we pre-registered a mixed effects model with implicit bias as the dependent variable, population density and proportion of Black people as covariates, a dummy for year as an additional covariate, a random intercept for county, and presence of a klavern (H1) or number of klaverns (H2) as the independent variable (https://osf.io/g3xru?view_only=cbf4ea078ad24bf09d0d8af89e1f3980). We estimated all multi-level models with the lmerTest package (Version 3.1.3; Kuznetsova et al., 2017). We obtained marginal effects using the marginaleffects package (Version 0.18.0; Arel-Bundock et al., forthcoming). Figure 1 shows the geographical distribution of implicit bias. Figure 2 shows the geographical distribution of Klan klaverns. Figure 3 shows the geographical distribution of White Supremacist activity.

Concerning H1, the pre-registered analysis shows a significant marginal^2^ effect of the presence of a Ku Klux Klan klavern on implicit bias (*b* = −0.005, *SE* = 0.002, *p* = .008), indicating lower levels of implicit bias in counties with a klavern compared with counties without a klavern. Concerning H2, the pre-registered analysis shows a significant marginal effect of the number of Ku Klux Klan klaverns in a county on implicit bias (*b* = −0.003, *SE* = 0.001, *p* < .001), indicating that counties with more klaverns show lower levels of implicit bias. Importantly, both analyses show effects in the opposite direction of our pre-registered hypotheses.

For H3 to H5, we pre-registered three analyses for each hypothesis: a linear regression, a spatial lag model, and an exploratory spatial error model (as described by Ebert et al., 2023), with White Supremacist activity as the dependent variable, population density and proportion of Black people as covariates, and implicit bias (H3), the presence of a klavern (H4), or the number of klaverns (H5) as the independent variable (https://osf.io/u3mbx/?view_only=8816ae9739134460ac071da5a5610615). Geographical data often have spatial dependencies: Geographical units that are closer together are more similar to each other. Spatial regressions can account for this clustering, whereas models that fail to account for these spatial dependencies can produce biased estimates (Anselin, 1988; Ebert et al., 2023).

To set up the spatial regressions, we followed the tutorial by Ebert and colleagues (2023). We first created a spatial weights matrix using k-nearest neighbor weights and used Moran scatter plots and Getis-Ord-Gi plots to assess spatial clustering and identify hot and cold spots. Next, we ran linear regressions with the above specifications to test our research questions and then tested for the presence of spatial autocorrelation of the residuals using Moran’s test. If there was no autocorrelation, we interpreted the linear regression. If there was spatial autocorrelation, we proceeded by running a spatial lag and a spatial error model (we pre-registered to use the spatial lag model as the main analyses). Finally, we used Moran’s test on the residuals of the spatial regressions to assess whether the spatial regressions adequately dealt with the spatial autocorrelation. We estimated all spatial regressions using the spatialreg package (Version 1.3.2, Bivand et al., 2013), aided by functionalities from the sf package (Version 1.0.14, Pebesma, 2018; Pebesma & Bivand, 2023).

For H3, the linear regression shows a significant marginal effect of implicit bias on White Supremacist activity on the county level (*b* = −16.5, *SE* = 3.62, *p* < .001). Moran’s test shows the presence of autocorrelation (Moran’s *I* = 28.953, *p* < .001). The spatial lag model shows a significant conditional effect of implicit bias on White Supremacist activity on the county level (*b* = −11.38, *SE* = 4.02, *p* = .004), whereas the spatial error model shows a non-significant effect (*b* = −7.16, *SE* = 3.98, *p* = .07). Again, these analyses all show effects in the opposite direction of the pre-registered hypotheses.

For H4, the linear regression shows a significant marginal effect of Ku Klux Klan presence on White Supremacist activity on the county level (*b* = 12, *SE* = 1.53, *p* < .001). Moran’s test shows the presence of autocorrelation (Moran’s *I* = 27.231, *p* < .001). Both the spatial lag (*b* = 9.43, *SE* = 0.73, *p* < .001) and the spatial error (*b* = 9.27, *SE* = 0.77, *p* < .001) show significant conditional effects of Ku Klux Klan presence on White Supremacist activity on the county level. These effects are in the same direction as the pre-registered hypotheses, indicating that counties with a klavern have more present-day White Supremacist activity than counties without a klavern.

For H5, the linear regression shows a significant marginal effect of the number of Ku Klux Klan klaverns on White Supremacist activity on the county level (*b* = 10.1, *SE* = 1.78, *p* < .001). Moran’s test shows the presence of autocorrelation (Moran’s *I* = 27.077, *p* < .001). Both the spatial lag model (*b* = 8.56, *SE* = 0.47, *p* < .001) and the spatial error model (*b* = 8.20, *SE* = 0.48, *p* < .001) show significant conditional effects of the number of the number of Ku Klux Klan klaverns on White Supremacist activity on the county level. These effects are in the same direction as the pre-registered hypotheses, indicating that counties with more klaverns also have more present-day White Supremacist activity.

### Robustness Analyses

Given the counter-intuitive nature of some of our findings, we present a range of robustness checks using a multiverse approach (see Primbs et al., 2022; Steegen et al., 2016). These robustness checks include (a) alternative analysis specifications, (b) alternative thresholds for bias-precision trade-offs, (c) alternative sets of covariates, (d) replications with other implicit measures, (e) discriminant tests, (f) replications with explicit measures, and (g) replications with representative datasets. We present a descriptive summary of the results for each robustness check here in the main text, and report the full statistical analysis results for each robustness check in the online supplementary materials (https://osf.io/h5sx6/?view_only=3f8cc0ff314f4410979f91e1b6f4be67).

#### Alternative Analysis Specifications

Each alternative analysis specification is aimed at targeting a potential weakness of the pre-registered main analyses. First, our covariates were strongly skewed. Therefore, we repeated several of our analyses with log-transformed covariates. Second, for H1 and H2 we had pre-registered a mixed effects model, but spatial regressions are more rigorous for use with geolocated data. Therefore, we tested H1 and H2 using the analyses we pre-registered for H3 to H5. Third, for H3 to H5, the dependent variable reflects the number of White Supremacist events and propaganda in a county, with a large number of countries having no or a very small number of events and propaganda in the period of 2017 to 2023, resulting in the variable being skewed. Furthermore, inspection of the residuals and predicted values of the Ordinary Least Squares (OLS) regression show common issues associated with modeling count data such as the prediction of impossible negative values and strong heteroskedasticity of the residuals, thus violating a number of assumptions (e.g., Ryan et al., 2021). We addressed these issues by conducting a GLM assuming a Poisson distribution, a Generalized Linear Model (GLM) assuming a negative binomial distribution, and zero-inflated variants of both.

Although Poisson regressions are commonly used for count data, the dependent variable also exhibited strong overdispersion, meaning that the variance is larger than the mean. In such instances, a Poisson regression is likely to result in false positives, so a negative binomial regression is more appropriate (Ryan et al., 2021). Furthermore, because a large number of counties have no White supremacy activity, we also specify zero-inflated models.

Table 1 provides a full overview over all frequentist models and results. Across all analysis specifications, we found no support for H1, the hypothesis that the presence of a Klan klavern in the past is associated with higher levels of present-day implicit bias. In contrast, across all model specifications, we found that the presence of a Klan klavern in the past was associated with lower levels of present-day implicit bias. Correspondingly, we did not also find support for H2, the hypothesis that a higher number of klaverns in a county is associated with higher levels of present-day implicit bias. Here, too, we found instead across all model specifications that a higher number of klaverns was associated with lower levels of implicit bias. Moreover, we also did not find support for H3, the hypothesis that present-day implicit bias is associated with higher levels of White Supremacist activity within counties. Instead, seven of nine models showed that higher levels of implicit bias were associated with lower levels of White Supremacist activity. Finally, we found support for H4 but not H5, the hypothesis that the presence (H4) or a higher number of klaverns (H5) is associated with higher levels of White Supremacist events. For H4, seven of nine model specifications supported the hypothesis, but for H5, only four out of nine model specifications supported the hypothesis.

**Table 1.** Summary of Alternative Models.

Hypothesis	Analysis	Covariates	Pre-registered	Coefficient^a^	*SE* ^b^	*p*
H1	Mixed effects with year dummy	Normal	Yes	–0.005^a^	0.002	.008
	Mixed effects	Normal	No	–0.004^a^	0.002	.019
	LM	Normal	No	–0.007^a^	0.003^b^	.010
	LM	Logged	No	–0.011^a^	0.003^b^	<.001
	Spatial lag	Normal	No	–0.006	0.003	.048
	Spatial error	Normal	No	–0.007	0.003	.037
	Spatial lag	Logged	No	–0.010	0.003	.003
	Spatial error	Logged	No	–0.010	0.004	.003
H2	Mixed effects with year dummy	Normal	Yes	–0.003^a^	<0.001	<.001
	Mixed effects	Normal	No	–0.003^a^	<.001	<.001
	LM	Normal	No	–0.006^a^	0.002^b^	<.001
	LM	Logged	No	–0.009^a^	0.002^b^	<.001
	Spatial lag	Normal	No	–0.005	0.002	.013
	Spatial error	Normal	No	–0.006	.002	.010
	Spatial lag	Logged	No	–0.008	.002	<.001
	Spatial error	Logged	No	–0.008	.002	<.001
H3	LM	Normal	Yes	–16.5^a^	3.62^b^	<.001
	Spatial lag	Normal	Yes	–11.379	4.015	.005
	Spatial error	Normal	Yes	–7.158	3.984	.072
	Poisson GLM	Logged	No	–29^a^	5.95^b^	<.001
	Negative binomial GLM	Logged	No	–28.8^a^	6.56^b^	<.001
	Zero inflated Poisson—count^c^	Logged	No	–54.2^a^	11.6^b^	<.001
	Zero inflated Poisson—zero	Logged	No	0.311^a^	0.135^b^	.022
	Zero inflated NB—count	Logged	No	–50.8	13.2	<.001
	Zero inflated NB—zero	Logged	No	–0.544	0.233	.019
H4	LM	Normal	Yes	12^a^	1.53^b^	<.001
	Spatial lag	Normal	Yes	9.426	0.727	<.001
	Spatial error	Normal	Yes	9.274	0.766	<.001
	Poisson GLM	Logged	No	5.35^a^	0.761^b^	<.001
	Negative binomial GLM	Logged	No	5.21^a^	0.78^b^	<.001
	Zero inflated Poisson—count	Logged	No	5.67^a^	0.905^b^	<.001
	Zero inflated Poisson—zero	Logged	No	–0.096^a^	0.019^b^	<.001
	Zero inflated NB—count	Logged	No	5.52^a^	0.867^b^	<.001
	Zero inflated NB—zero	Logged	No	0.018^a^	0.027^b^	.514
H5	LM	Normal	Yes	10.1^a^	1.78^b^	<.001
	Spatial lag	Normal	Yes	8.556	0.469	<.001
	Spatial error	Normal	Yes	8.202	0.484	<.001
	Poisson GLM	Logged	No	0.475^a^	1.63^b^	.771
	Negative binomial GLM	Logged	No	596^a^	758^b^	.455
	Zero inflated Poisson—count	Logged	No	0.646^a^	0.312^b^	.038
	Zero inflated Poisson—zero	Logged	No	–0.089^a^	0.017^b^	<.001
	Zero inflated NB—count	Logged	No	685^a^	1,163^b^	.556
	Zero inflated NB—zero	Logged	No	0.029^a^	0.021^b^	.164

*Note.* The covariates included are population density and the proportion of Black people in a county (unless otherwise specified).

a These coefficients represent the average marginal effect. Otherwise, the coefficient represents the regression coefficient, which can generally be interpreted as a marginal effect for linear models without interactions. We only present the regression coefficient if the calculation of the average marginal effect is not possible in R. This is the case for all spatial regression models. ^b^These standard errors represent a heteroscedasticity-robust HC3 standard error. ^c^Zero-inflated models are two-component mixture models and return two coefficients, a count model coefficient and a zero-inflation model coefficient. We report both coefficients here.

In addition, we investigated whether we find statistical support for an effect of White Supremacist activity on implicit bias, as relationships in both directions are theoretically plausible. We ran a linear regression with standard covariates, a linear regression with logged covariates, a spatial lag model with standard and logged covariates, and a spatial error model with standard and logged covariates. All analyses were significant and showed that higher levels of White Supremacist activity were associated with lower levels of implicit bias. The full results can be inspected in the online supplementary materials (https://osf.io/h5sx6/?view_only=3f8cc0ff314f4410979f91e1b6f4be67).

Finally, we conducted a Bayesian spatial regression assuming a Poisson and negative binomial distribution, specifying spatial lag models. Our choice for a Bayesian model is guided by the available software (INLA, Version 24.2.9; Rue et al., 2009) and currently does not support spatial error models and zero-inflated models. Considering H3, accounting for spatial autocorrelation does not alter the direction of the effect and the credibility interval does not contain zero, indicating a significant negative effect of regional bias on White supremacy activity, consistent with the other models. Considering H4 and H5, accounting for spatial autocorrelation does not alter the direction of the effect and the credibility interval does not contain zero, indicating a significant positive effect of klavern presence or number on White Supremacist activity, consistent with the other models. Table 2 shows an overview of the Bayesian analyses.

**Table 2:** Overview of Bayesian Analyses.

Hypothesis	Analysis	Covariates	Coefficient	*SE*	Credibility interval
H3	Spatial Poisson	Logged	–3.172	0.491	[–4.135, –2.208]
	Spatial NB	Logged	–3.179	0.490	[–4.140, –2.217]
H4	Spatial Poisson	Logged	0.644	0.066	[0.514, 0.773]
	Spatial NB	Logged	0.643	0.066	[0.514, 0.773]
H5	Spatial Poisson	Logged	0.356	0.040	[0.277, 0.435]
	Spatial NB	Logged	0.356	0.040	[0.277, 0.435]

### Inclusion-Precision Trade-Offs

When analyzing geographical data, researchers should consider inclusion-precision trade-offs. In the context of the present research, we considered whether to include as many counties as possible in our analyses or for county means to be estimated with high degree precision. The downside of including as many counties as possible is that some counties contain relatively few responses, so mean estimates from those counties may be noisy or imprecise. In contrast, the downside of estimating means with a high degree of precision is that excluding counties with relatively few responses necessarily excludes rural areas, thereby biasing the remaining sample toward urban areas.

In our main analyses, we included all counties for which we have data. To assess the robustness of our findings against this analytic decision, we conducted additional analyses with counties that have at least 100 or 200 observations. We repeat all analyses described in Table 1, with the exception of the mixed models.^3^ Across almost all analysis specifications, we find results consistent with the findings presented in Table 1 for all hypotheses. In addition, we find that White Supremacist activity negatively predicts implicit bias scores. Our findings are therefore robust to different inclusion-precision trade-off thresholds.

### Alternative Covariate Specifications

The pre-registered set of control variables represents one out of many possible sets of appropriate control variables. Therefore, we conducted an additional set of analyses including two more covariates: education and income. Education was operationalized as the proportion of people over 25 with at least a high school degree in a county. Income was operationalized as the median income per household in a county. We repeated the analyses specified above (Table 1, but without mixed models) including these two additional covariates for the full dataset, for counties with at least 100 observations, and for counties with at least 200 observations. Across all analyses specifications, we again find results consistent with the findings presented in Table 1, and also that White Supremacist activity negatively predicts implicit bias.

### Replication: Skin Tone Bias

We replicated our main analyses using data from the Skin Tone IAT. The Skin Tone IAT assesses evaluations of light skin relative to dark skin. As the Ku Klux Klan frequently targeted Black people, it therefore targeted people with a relatively darker skin color. Therefore, our main findings based on implicit bias toward Black people should also hold for implicit bias toward people with dark skin. We therefore repeated all analyses reported in Table 1 on data from the Skin Tone IAT of Project Implicit. We used the 2004 to 2023 subset and removed all participants with missing IAT scores, all participants without location information, and all participants who have 10% or more responses faster than 300 ms on the IAT. The final sample included 890,148 participants, of whom 612,930 live in counties where there was a klavern and 277,218 live in counties where there was no klavern. We find that the results are directionally consistent with the findings presented in Table 1. However, for H1, some of the results are just shy of the traditional threshold for statistical significance, with two analyses *p* < .05 and four analyses with *p* < .1. For H2, all analyses are statistically significant at *p* < .05. We therefore conclude that the presence or number of klaverns in a county is associated with lower levels of implicit skin tone bias, but that this association is less robust than for the race bias. For H3, we find across eight of nine analysis specifications that lower levels of implicit skin tone bias predict more White Supremacist activity. H4 and H5 do not include implicit bias data and are thus not further considered here. Finally, we find that White Supremacist activity negatively predicts implicit skin tone bias.

### Discriminant Tests: Age and Disability Bias

The Ku Klux Klan explicitly targeted particular social groups, including Black people, their allies, and other racial and religious minority groups. Therefore, the historical and cultural legacy of the Klan should be limited to groups targeted by the Klan. We test this idea by repeating our analyses on the Age IAT and the Disability IAT from Project Implicit. The Age IAT assesses evaluations of young relative to old people. We used the 2002 to 2021 subset of the Age IAT from Project Implicit. The pre-processing was identical to the pre-processing of the Skin Tone IAT. The final sample included 934,526 participants, 629,762 of whom live in counties where there was a klavern and 304,674 live in counties where there was no klavern. The disability IAT assesses evaluations of abled relative to disabled people. We used the 2004 to 2021 subset of the disability IAT from Project Implicit. The pre-processing was identical to the processing for the Skin Tone IAT. The final sample included 418,181 participants, 283,665 of whom live in counties where there was a klavern and 134,516 live in counties where there was no klavern. Considering H1 and H2, zero analysis specifications found a significant correlation between the presence or number of klaverns and either implicit age or disability bias. Considering H3, the count models often showed significant correlations between implicit age and disability bias and White Supremacist activity today. However, these correlations vary in different directions, depending on the specified count model, thus showing opposite patterns. Moreover, the spatial models always find no significant correlation between implicit bias and White Supremacist activity. We therefore argue that findings of the count models may constitute false positives that are potentially caused by spatial autocorrelation, which can result in false positives if not adequately accounted for (Anselin, 1988; Ebert et al., 2023). This interpretation is further bolstered by analyses in which we reversed implicit bias (so that it is the DV) and White Supremacist activity (so that it is the IV). This specification allowed us to circumvent the problems associated with the analyses of spatially dependent count data with zero inflation. For age bias, zero analyses show a significant effect. For disability bias, four of six analyses show a significant correlation between White Supremacist activity and implicit disability bias.

We conclude that these data provide no consistent evidence for an association between Ku Klux Klan activity and implicit age or disability bias, providing an important test of discriminative validity. However, these data suggest a relationship between modern-day White Supremacist activity and implicit disability (but not age) bias. This pattern of results might be driven by the fact that modern-day White Supremacists also hold strong biases against disabled people (see, e.g., the infamous video of Donald Trump mocking a disabled reporter).

#### Replication: Explicit Race Bias

Though Payne and colleagues’ (2017) situational model of bias focuses on implicit bias, other perspectives (e.g., Calanchini et al., 2022) include analyses of explicit biases to further strengthen the empirical basis of their claims. Therefore, we repeated all analyses specified above with explicit racial bias instead of implicit racial bias as a variable of interest. Explicit bias was operationalized as the difference between two feeling thermometers. Higher scores mean that people felt warmer about White compared with Black Americans. Participants were asked to rate how warm or cold they feel toward White, or separately, Black people on an 11-point Likert-type scale ranging from “*Extremely Cold*” to “*Extremely Warm*.” Because Project Implicit changed the scale of the feeling thermometer between years, we only used the subset of the data from 2016, the year of the latest change, to 2022. For H1 to H3, we replicated the results observed in Table 1, such that there was a negative effect of Klan presence or number of Klaverns on regional average explicit bias, and a negative effect of regional average explicit bias on modern White Supremacist activity. H4 and H5 do not include racial bias as a variable and were not further investigated. We conclude that our findings also extend to explicit racial bias.

#### Replication: Representative Dataset

We further attempted to replicate our findings using a representative dataset: The Nationscape data (Tausanovitch & Vavreck, 2021). Whereas participants in the Project Implicit datasets are people who visit the website and self-select an IAT test, the Nationscape dataset contains participants recruited to be more or less representative of the U.S. population. The Nationscape dataset was a survey collected between July 2019 and January 2021 about topics and issues in the 2020 American national election. The data include questions about perceptions of social groups, racism, and geographical data at the congressional district level. Perceptions of social groups were measured with the question: “Here are the names of some groups that are in the news from time to time. How favorable is your impression of each of these groups or haven’t you heard enough to say?.” This question was asked about a variety of target groups, including “Whites” and “Blacks,” and was answered on a scale from *“(1) Very favorable”* to *“(4) Very unfavorable.”* We calculated the difference score between both target groups. Higher scores again mean that people rated White Americans more favorable compared with Black Americans. The dataset features 292,922 White participants who completed all relevant measures and for whom was geographical information available, 247,298 in congressional districts with a klavern and 45,624 in congressional districts without a klavern. Note that no county-level data were available. The Klan data included longitude and latitude, but the White Supremacist activity data did not include precise measures of geography, so we only tested H1 and H2 in this representative sample. We want to note explicitly that the representativeness of the sample hinges on the inclusion of the appropriate survey weights. We included the survey weights for all linear models. We did not include the survey weights for the spatial regressions, as the available software does not allow for the inclusion of multiple sets of weights in one analysis (i.e., for both representativeness and spatial dependence). We analyzed these data in the same way for H1 and H2 in the previous robustness checks, with the difference score between the perceived favorability of White and Black people as the dependent variable. For H1, the presence of a klavern, we find that all effects are in the same direction as in Table 1, but only one of six is statistically significant. For H2, the number of klaverns, we find that all effects are in the same direction as in Table 1, and four of six analyses are statistically significant, including all analyses weighted to be representative. We therefore conclude that our findings partially replicate in a representative sample.

## Discussion

In this article, we investigated whether the historical and cultural legacy of the Ku Klux Klan persisted within geographical regions. We found that, contrary to our predictions, both the presence of a klavern (H1) and the number of klaverns (H2) within counties were associated with lower levels of implicit racial bias. Moreover, also contrary to our predictions, we found that higher levels of implicit bias were associated with lower levels of modern White Supremacist activity (H3). Finally, and in line with our predictions, we found that the presence of a klavern (H4) and the number of klaverns (H5) were associated with more modern White Supremacist activity. We generally replicated this pattern of effects across many analysis specifications, for other IATs, for explicit measures, and in a representative dataset.

We embedded our research in situational models of bias: Historical inequalities are reflected in present-day implicit bias (Payne et al., 2017, 2019; Vuletich et al., 2024). As such, the Bias of Crowds model (Payne et al., 2017; Payne & Hannay, 2021) predicts that higher levels of historical inequalities should be associated with higher levels of implicit racial bias. Yet, we found evidence for the opposite: Higher levels of historical inequalities in the form of more Ku Klux Klan activity were associated with lower levels of modern implicit bias. Likewise, higher levels of discriminatory behavior should also be associated with higher levels of implicit bias. Yet, we find that more White Supremacist activity was predicted by, and predicted, lower levels of implicit racial bias. Interestingly, and in line with our predictions, analyses linking historical inequalities directly to modern behavior show that more inequality in the past is associated with more biased behavior today. Our findings are therefore largely incompatible with a Bias of Crowds perspective and require re-orienting current thinking about historical inequalities and racial bias.

### Selection Biases and Project Implicit

A possible explanation for our unexpected findings is selection effects, such that results depend on specific participant samples. Past research has highlighted the role of selection effects and argued that some findings may be more parsimoniously explained by changes in participant sample characteristics than by actual changes in bias (Primbs, Connor, et al., 2024; Primbs, Holland, et al., 2026). If we apply this thinking to the present case, people with more positive racial attitudes who live in areas with more reminders of past Ku Klux Klan activity and more exposure to present-day White Supremacist activity might be more likely to visit Project Implicit. In turn, these between-county differences in who visits Project Implicit might account for the observed counter-intuitive findings. However, past research using this same Project race IAT data to test similar hypotheses derived from the Bias of Crowds perspective has found predicted effects, such that regions with a greater reliance on slavery in 1860 also showed higher levels of implicit bias (Payne et al., 2019). Moreover, we partially replicated our main findings in a different dataset (i.e., Nationscape) that does not rely on participants who choose to visit the Project Implicit demonstration website. Thus, selection bias is unlikely to explain our findings.

### Historical Oppression Engenders Positivity

In contrast to our initial predictions based on the Bias of Crowds perspective, we now propose instead that a more parsimonious explanation for our findings is that both historical and present-day oppressions can engender implicit positivity (Kurdi et al., 2023). Whereas traditional perspectives on intergroup bias conceptualize social group evaluations as simple associative links, Kurdi and colleagues instead adopt a propositional perspective in which such links contain relational information. Thus, environmental reminders of inequality connect the oppressed group (Black people) with positive evaluations because they are victims of negative treatment, and connect the oppressing group (White people) with negative evaluations because they are the perpetrators of negative treatment. This explanation is in line with a series of seven experiments showing that exposing U.S. Americans to narratives about oppression increases positive evaluations of the oppressed group (Kurdi et al., 2023). The historical and cultural legacy of the Ku Klux Klan and modern White Supremacist activity would seem to constitute such reminders of oppression, which in turn could create implicit positivity toward oppressed groups.

Our research therefore advances our understanding of the impact of social and cultural environments on implicit bias: The presence of historical and modern inequalities in our daily environments is not necessarily linked to greater levels of implicit bias against oppressed groups. Instead, reminders of inequality can engender implicit positivity toward oppressed groups (Kurdi et al., 2023). Consequently, our findings suggest that the simple concept of accessibility as the mechanism proposed to underlie the Bias of Crowds model is not sufficient to explain the effects of historical inequality on implicit bias. To reconcile our finding that historical Klan locations are negatively related to modern biases with Payne and colleagues’ (2019) findings that historical slave populations are positively related to modern biases, future research should examine more historical inequalities and biases other than race to clarify the conditions under which historical oppression engenders positivity versus negativity.

### The KKK and the Public Conscience

Another possible explanation compatible with both situational models and Kurdi and colleagues’ (2023) perspective is that historical KKK activity strongly highlighted regional racial divides and conflicts. The extremity of the KKK’s positions and actions might have pushed moderate and liberal White toward more egalitarian social attitudes as they sought to distance themselves from the KKK. These more egalitarian attitudes may have persisted across time within regions and are reflected in implicit bias measures today. Such explanations are in line with research that shows that attitudes that are more strongly present in the public conscience are more likely to change compared with attitudes less in the public conscience (Charlesworth & Banaji, 2019). In contrast, moderate and liberal White people in regions without historical KKK activity had less direct contact with the KKK and their actions and in turn did not undergo this shift in attitudes. These unaltered attitudes may have also persisted across time within regions, are picked up by our measures today, and hence create the observed pattern of effects.

### Constraints on Generalizability

In the present research, we identified relationships among historical Klan locations, modern White Supremacist activity, and current biases that are robust against a variety of analytic decisions. However, considering the complexity of the causal net at play and the already very large number of robustness checks, we have decided against attempting to further establish causality in the current article beyond what is already implied by the temporal constraints of these data (i.e., historical Ku Klux Kan activity may influence modern biases, but not vice versa), instead leaving this task to future research. Moreover, our research mostly relies on data from Project Implicit, a largely self-selected online sample, and our replication in a representative dataset showed less consistent patterns of statistical significance. Hence, the observed effects may be weaker in the general population. Finally, we limited ourselves to one particular hate group: The Ku Klux Klan. However, neither the Klan nor the United States holds the monopoly on hate. Other similar groups have operated, and continue to operate, in a variety of racial, ethnic, religious, and other contexts around the world. Future research should continue to investigate the relationship between legacies of hate and modern biases and further test our predictions about historical oppression engendering positivity.

## Conclusion

Taken together, our research advances situational models of implicit bias by showing that historical legacies of hate can have counter-intuitive consequences and cause positive racial attitudes toward oppressed group members. Thus, current models of the relationship between historical inequalities and implicit bias may be overly simplified. That said, the legacy of the Ku Klux Klan is neither positive nor desirable. Instead, our findings illustrate that “even in the darkest of nights I see the moonlight” (Bloodborne, 2015), and even a legacy of hate and terror can spawn hope.

## Notes

1. There were three comings of the Ku Klux Klan. The second Klan featured the most members.

2. A marginal effect is the effect in the population. A conditional effect is the effect in a particular stratum. Further information on marginal and conditional effects can be found here: https://www.the100.ci/2022/05/27/%E2%9C%A8-unleash-your-inner-stats-sparkle-%E2%9C%A8-with-this-very-non-technical-introduction-to-marginal-effects/

3. We view the mixed models as inferior to the spatial regressions for analyzing regional data and therefore conduct spatial regressions where possible.
